# Aldh1-Expressing Endocrine Progenitor Cells Regulate Secondary Islet Formation in Larval Zebrafish Pancreas

**DOI:** 10.1371/journal.pone.0074350

**Published:** 2013-09-17

**Authors:** Hiroki Matsuda, Michael J. Parsons, Steven D. Leach

**Affiliations:** Department of Surgery and the McKusick-Nathans Institute of Genetic Medicine, Johns Hopkins University, Baltimore, Maryland, United States of America; National Institutes of Health / NICHD, United States of America

## Abstract

Aldh1 expression is known to mark candidate progenitor populations in adult and embryonic mouse pancreas, and Aldh1 enzymatic activity has been identified as a potent regulator of pancreatic endocrine differentiation in zebrafish. However, the location and identity of Aldh1-expressing cells in zebrafish pancreas remain unknown. In this study we demonstrate that Aldh1-expressing cells are located immediately adjacent to 2F11-positive pancreatic ductal epithelial cells, and that their abundance dramatically increases during zebrafish secondary islet formation. These cells also express *neurod*, a marker of endocrine progenitor cells, but do not express markers of more mature endocrine cells such as *pax6b* or *insulin*. Using formal cre/lox-based lineage tracing, we further show that Aldh1-expressing pancreatic epithelial cells are the direct progeny of pancreatic notch-responsive progenitor cells, identifying them as a critical intermediate between multi-lineage progenitors and mature endocrine cells. Pharmacologic manipulation of Aldh1 enzymatic activity accelerates cell entry into the Aldh1-expressing endocrine progenitor pool, and also leads to the premature maturation of these cells, as evidenced by accelerated *pax6b* expression. Together, these findings suggest that Aldh1-expressing cells act as both participants and regulators of endocrine differentiation during zebrafish secondary islet formation.

## Introduction

In mammals, pancreatic endocrine differentiation occurs through a defined sequence of progenitor cell types, which undergo progressive lineage restriction [1-3]. Detailed elaboration of this step-wise differentiation program has allowed progress towards the guided programming of human stem cells towards a β-cell fate [4], potentially providing a source of insulin-producing cells suitable for cell replacement therapy in diabetes.

Complementing these mammalian studies, the zebrafish (*Danio rerio*) has emerged as a highly productive model organism in which to study mechanisms of pancreas development and β-cell differentiation, with multiple studies demonstrating that fish and mammals rely on shared transcriptional and signaling networks during pancreas development. Among these shared mechanisms are multiple transcription factors, including *pdx1, isl1, mnx1, neurod, nkx2.2, nkx6.1, pax6, prox1, sox9b, hnf1α, hnf1β, hnf3β*, and *hnf3γ*, which appear to have similar functions in developing mouse and zebrafish pancreas [5-18]. However, inter-species differences also exist in the pancreatic developmental programs employed in mammals and in zebrafish. Among these differences is the presence of an early appearing principle islet in zebrafish, which arises from a *ptf1a*-independent lineage that is both temporally and spatially segregated from progenitor cells destined to give rise to the exocrine pancreas [13,14,19].

In contrast to developmental programs responsible for formation of the principle islet, secondary islet formation in zebrafish appears to occur in a manner more similar to that observed during mammalian islet formation. As in mammals, notch-responsive cells associated with developing zebrafish ductal epithelium generate endocrine progenitor cells that delaminate and proliferate prior to differentiating and coalescing to form organized islets [10,20]. These zebrafish pancreatic notch-responsive cells (PNCs) have been implicated as precursors for α, β, and δ endocrine lineages, and are subject to precocious endocrine differentiation following pharmacologic or genetic inhibition of notch signaling [10,12,20,21]. However, the intermediate cell states associated with generation of mature endocrine cells from PNCs remain incompletely characterized.

In addition to notch, recent studies have also implicated retinoic acid signaling in the regulation of endocrine differentiation during zebrafish secondary islet formation. While Aldh1 enzymatic activity and associated retinoic acid production are known to exert an early positive influence on the pancreatic progenitor field [22-24], they have recently been shown to exert a negative influence on β-cell differentiation during secondary islet formation [25]. In addition to this functional data in zebrafish, Aldh1 isoform expression and enzymatic activity have also been utilized to mark candidate progenitor populations in murine pancreas [26,27]. Using a variety of transgenic lines to mark pancreatic cell types and lineages in embryonic and larval zebrafish, we have confirmed the presence and identity of Aldh1-expressing progenitor cells during zebrafish secondary islet formation, and have further placed these cells within a multi-step endocrine differentiation paradigm. Using both Cre/lox lineage tracing and pharmacologic manipulation, we further demonstrate that Aldh1 enzymatic activity regulates both entry and exit from the Aldh1-expressing progenitor compartment. These results identify Aldh1-expressing cells as both a participant and a regulator of endocrine differentiation during zebrafish secondary islet formation.

## Materials and Methods

### Zebrafish strains

All experiments involving zebrafish were approved by the Johns Hopkins University Institutional Animal Care and Use Committee. Fish were raised and maintained under standard laboratory conditions. Euthanasia was accomplished by immersion in 0.16% tricaine followed by placement in an ice bath, consistent with recommendations of the Panel on Euthanasia of the American Veterinary Association. We used the following transgenic lines: *TgBAC*(*ptf1a:eGFP*)^jh1^ [28]; *Tg*(*pax6b:eGFP*)^*ulg515*^ [16]; *Tg*(*-2.6mnx1:GFP*)^*ml5*^ [17]; *TgBAC*(*neurod:eGFP*)^*nl1*^ [29] *Tg*(*kdrl:GRCFP*)^*zn1*^ [30]; *Tg*(*Tp1bglob:hmgb1-mCherry*)^*jh11*^ [10]; *Tg*(*Tp1bglob:eGFP*)^*um14*^ [10]; (*Tp1glob:creERT2*)^*jh12*^ [21]; *Tg*(βactin*:loxP-stop-loxP-hmgb1-mCherry*)^*jh15*^ [21].

### Immunohistochemistry

For immunofluorescent labeling, pancreatic tissue was fixed in 4% paraformaldehyde/phosphate buffered saline(PBS) at 4°C overnight and immersed in 30% sucrose/PBS, as previously described [10,12,31]. Tissue was then embedded in optimal cutting temperature (OCT) compound, frozen in liquid nitrogen, and sectioned at 10 µm using a cryostat. After permeabilization with PBS-containing 0.2% Triton X-100, sections were treated with 10% FBS in PBS-containing 0.1% Triton X-100 for blocking and incubated with primary antibody diluted with 1% FBS in PBS-containing 0.1% Triton X-100 (PBSTx). The following primary antibodies were utilized at the indicated dilution: rabbit anti-Aldh1a (Abgent) at 1:50, guinea pig anti-insulin (Abcam) at 1:300, monoclonal mouse anti-eGFP (Invitrogen) at 1:1000, and monoclonal mouse anti-2F11(Abcam) at 1:1000. After three washes with PBSTx, sections were incubated with AlexaFluor-conjugated secondary antibodies (Invitrogen) at 1:500-1000 dilution with PBSTx.

For whole mount immunofluorescent staining, 5 dpf larva were fixed in 4% paraformaldehyde/phosphate buffered saline(PBS) at 4°C overnight and dehydrated with a methanol series at room temperature. Tissues were rehydrated to PBSTx, treated with proteinase K (25 µg/ml) for 25 min, refixed with 4% paraformaldehyde in PBSTx for 20 min, treated with blocking buffer (10% donkey serum, 1% DMSO, 0.1% Triton X-100 in PBS), and incubated with either rabbit anti-Aldh1a (1:50) (Abgent) or monoclonal mouse 2F11 (1:100) (Abcom) in blocking buffer at 4°C overnight. After washing with PBSTx, samples were incubated with AlexaFluor conjugated secondary antibodies (Invitrogen) at a 1:500 dilution with blocking buffer.

Fluorescent images were acquired with Nikon A1 scanning confocal microscope, and areas and volumes of labeling for specific markers were calculated using NIS-Elements software.

### FACS sorting

Pancreatic tissue was dissected from adult AB and *Tg*(*TP1bglob:eGFP*) fish, incubated in 0.5% bleaching solution for 2 min and digested in 1.4 mg/ml collagenase-P at 37 °C for 20 min. Following multiple washes with HBSS supplemented with 5% FBS, collagenase-digested pancreatic tissue was filtered through 500 µm polypropylene mesh (Spectrum Laboratories), then spun through a 30% FBS cushion. For FACS, pelleted-pancreatic tissue were resuspended in diluted trypsin (0.05%) (Invitrogen) and incubated at 37 °C for 15 min. Dispersed cells from AB and Tg(TP1bglob:eGFP), were then directly resuspended in Aldefluor buffer and 0.8 x PBS, respectively. To label Aldh1-positive cell for FACS-sorting, the Aldefluor Kit (Stem Cell Technologies) was utilized according to the manufacturer’s protocol. Flow cytometry was performed using a FACSAria (Becton Dickinson) flow cytometer.

### RT-PCR

Gene expression in FACS-sorted TP1bglob:eGFP+ cells and Aldefluor+ cells was analyzed by RT-PCR. Total RNA was extracted from FACS sorting cells (Aldeflour-, Aldefluor+, Tp1-, TP+) using the RNeasy Micro Kit (QIAGEN). cDNA was synthesized with Omniscript RT Kit (Qiagen) according to the manufacturer’s protocol. RT-PCR was performed with Apex Taq RED Master Mix,2.0X (Genesee Scientific) with 30 ng cDNA as templates. The following primer sequences were utilized: Aldh1a2 forward primer (aaccactgaacacggacctc) and reverse primer (cgtcttgcctgacatcttca); β-actin forward primer (taccccattgagcacggtat) and reverse primer (cggtcaggatcttcatcagg); prom1 (cd133) forward primer (tgaggaggctggtgaagagt) and reverse primer (cctgcagagtctccaaggtc); sca1 forward primer (atcgcagagaggtgctgaat) and reverse primer (gcaggagaaacttggctgtc); elastaseA forward primer (atcaagttggctgagcctgt) and reverse primer (ccttgtcaacccagtcagtg); krt18 forward primer (tttcccagatcatggaggag) and reverse primer (tcgtactcctgcgtctgatg); insulin forward primer (ctgttggtcgtgtccagtgt) and reverse primer (ggagagcattaaggcctgtg); e-cad forward primer (gacgttggaaggaagagctg) and reverse primer (agagcgtccttgttcttcca).

### Cell labeling with EdU

The Click-iT EdU Alexa Fluor647 Imaging Kit (Invitrogen) was utilized according to the manufacturer’s protocol. Larvae were incubated in 1 mM EdU/1 %DMSO in embryo medium for 2 hours, fixed in 4% PFA in PBS at 4°C overnight, immersed in 30% sucrose/PBS, embedded in OCT compound, frozen in liquid nitrogen, and sectioned at 10 mm using a cryostat. After EdU staining according manufacturer’s protocol, additional immunostaining was performed. The percentage of EdU+ cells in relevant tissue compartments was then determined at 15, 20 and 25dpf, using 10 fish at each developmental stage and a minimum of 3 pancreatic sections per fish.

### Temporal control of CreERT2 activity

4-Hydroxytamoxifen (4OHT, T176, Sigma) treatment were performed at 3-5 dpf and 18-20 dpf as previously described [21].

## Results

### Aldh1-positive cells progressively accumulate during zebrafish secondary islet formation

**Figure 1:**
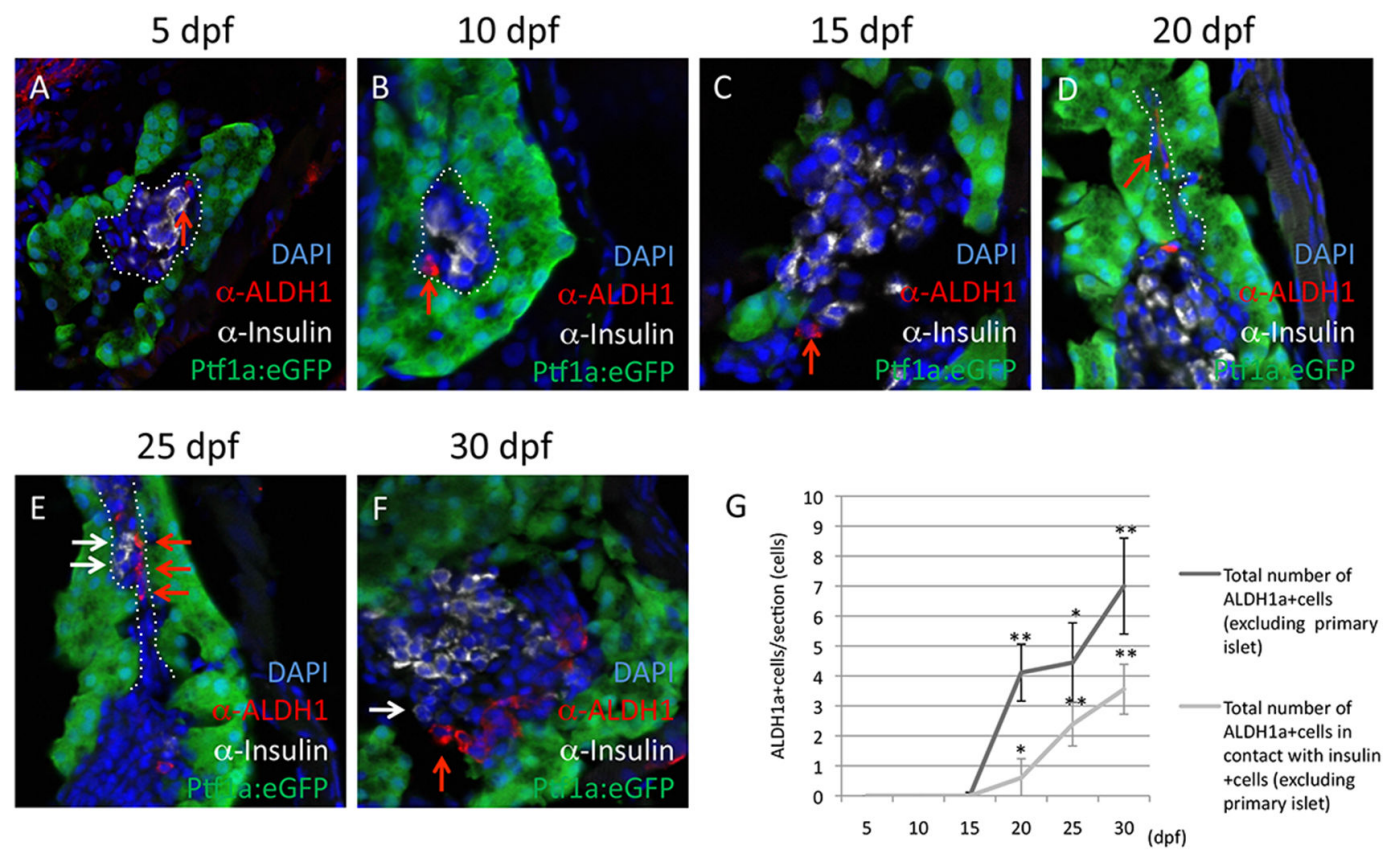
Aldh1-expressing cells increase in number and are spatially associated with endocrine cells during secondary islet formation. Expression of Aldh1 (red arrows) is compared with that of Insulin (white arrows), as assessed immunofluorescent labeling at 5 (A), 10 (B), 15 (C), 20 (D), 25 (E) and 30 dpf (F). (G) Quantification of the number of Aldh1-expressing cells located outside of the principle islet (black line), and their juxtaposition with Insulin-expressing cells (grey line). Values were derived from n=10 fish per developmental time point, with a minimum of 3 sections per fish. (*) and (**) indicate p<0.05 and p<0.01, respectively. Dotted white lines in (A) and (B) indicate region of principle islet; dotted white lines in (D) and (E) indicate region of pancreatic duct.

In order to evaluate the presence and possible function of Aldh1-expressing cells during secondary islet formation in larval zebrafish pancreas, we first surveyed Aldh1 expression in conjunction with other lineage markers. As previously reported, secondary islet formation in zebrafish larvae occurs between 5 and 20 dpf, with approximately 50% of larvae having *pax6b*-expressing secondary islet tissue by 10dpf, and essentially 100% of larvae having discernible secondary islet tissue by 20 dpf [10]. Between 5-15 dpf, pancreatic Aldh1 immunoreactivity was confined to the principle islet, where Aldh1-positive cells were often observed in proximity to Insulin-positive β-cells (Figure 1A-C). By 20 dpf, small numbers of Aldh1-positive cells also became detectible outside of the principle islet. These cells were frequently observed in association with forming ductal epithelium, and were uniformly negative for ptf1a:eGFP, a marker of exocrine cells (Figure 1D-F). They were also negative for Insulin as assessed by double immunofluorescent labeling, but were frequently observed to be immediately adjacent to Insulin-positive cells (Figure 1E). When quantified as the total number of cells per section (n=10 fish with minimum 3 pancreatic sections per fish), non-principle islet associated Aldh1-positive cells were found to steadily increase between 15 and 30dpf (Figure 1G). Beginning at 20 dpf, a similar steady increase in the number of non-principle islet associated Aldh1-positive cells in direct contact with Insulin-positive cells was also observed, raising the question of whether these duct-associated Aldh1-positive cells might represent a population of β-cell precursors.

### Aldh1-positive cells express markers of endocrine progenitor cells

**Figure 2:**
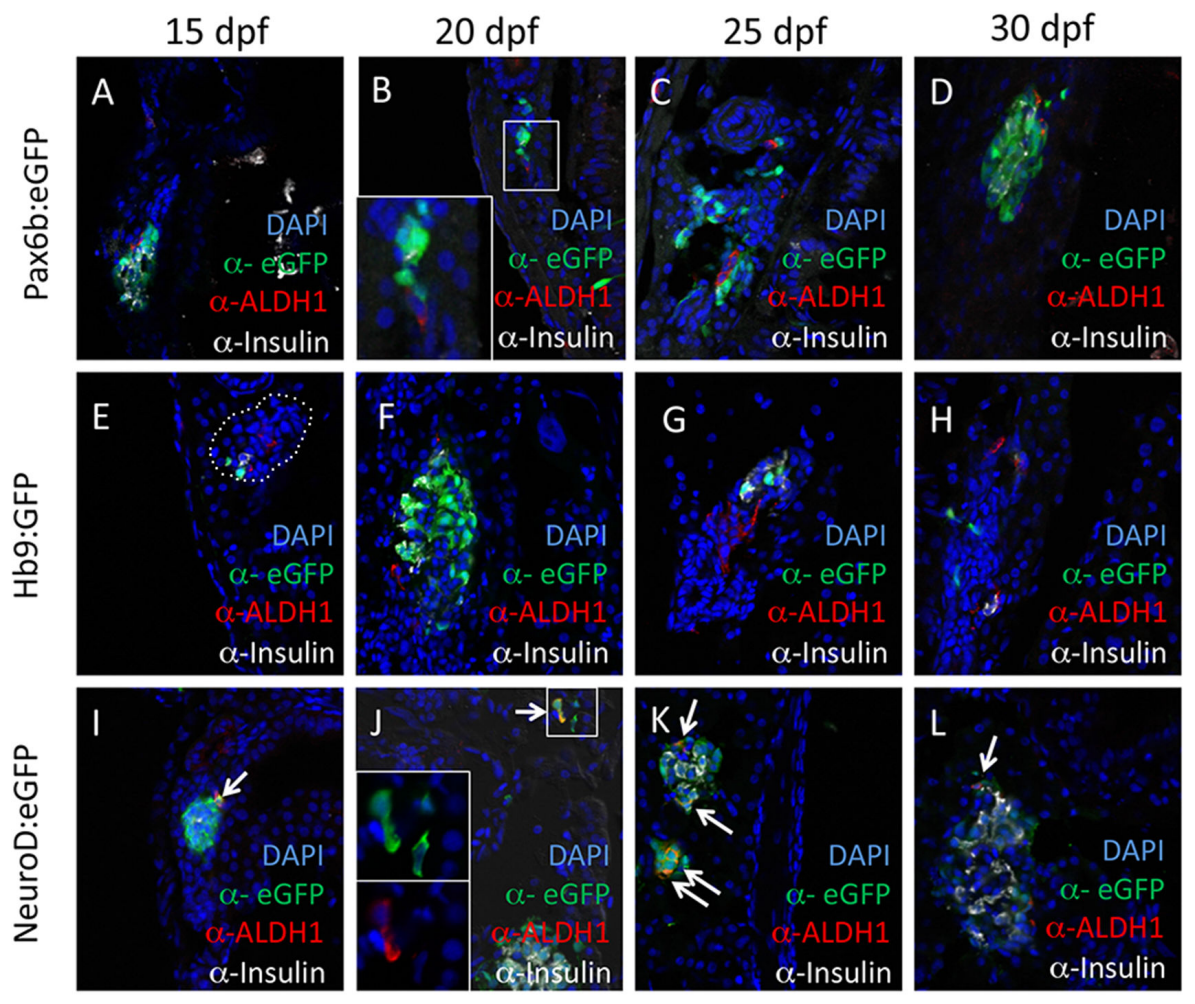
Aldh1 is expressed in NeuroD^pos^ endocrine progenitor cells. Expression of Aldh1 is compared with that of Pax6b:eGFP (pan-endocrine cell marker; A-D), Hb9:eGFP (early β cell marker; E-H) and NeuroD:eGFP (endocrine progenitor marker; I-L) at 15 (A, E and I), 20 (B, F and J), 25 (C, G and K) and 30 dpf (D, H and L). Note that Aldh1 is co-expressed with eGFP in NeuroD:eGFP fish (arrows), but not in Pax6b:eGFP and Hb9:eGFP fish. Dotted white lines in (E) indicate region of principle islet.

To further characterize Aldh1-positive cells in larval zebrafish pancreas, we examined Aldh1 expression in conjunction with additional endocrine markers. These included *Tg*(*pax6:GFP*)^*ulg515*^ (hereafter referred to as pax6b:GFP), a pan-endocrine marker [16], *Tg*(*-2.6mnx1:GFP*)^*ml5*^ (hereafter referred to as hb9:GFP), a marker of early β-cells [32]; and *TgBAC*(*neurod:eGFP*)^*nl1*^, (hereafter referred to as neurod:eGFP), a marker of endocrine progenitor cells as well as differentiated endocrine cells [33]. These analyses revealed that, between 15 and 30dpf, Aldh1 immunoreactivity marked cells also labeled with neurod:eGFP, but not hb9:eGFP or pax6b:GFP (Figure 2A-L). While many Aldh1-negative, *neurod*-positive cells were identifiable within islet tissue, virtually all Aldh1-positive cells also expressed neurod:eGFP. Because neurod:eGFP is felt to label cells undergoing early commitment to the endocrine lineage, these findings suggest that Aldh1 is transiently expressed in at least a fraction of early endocrine progenitor cells.

### Aldh1-positive endocrine progenitor cells are associated with ductal epithelium

**Figure 3:**
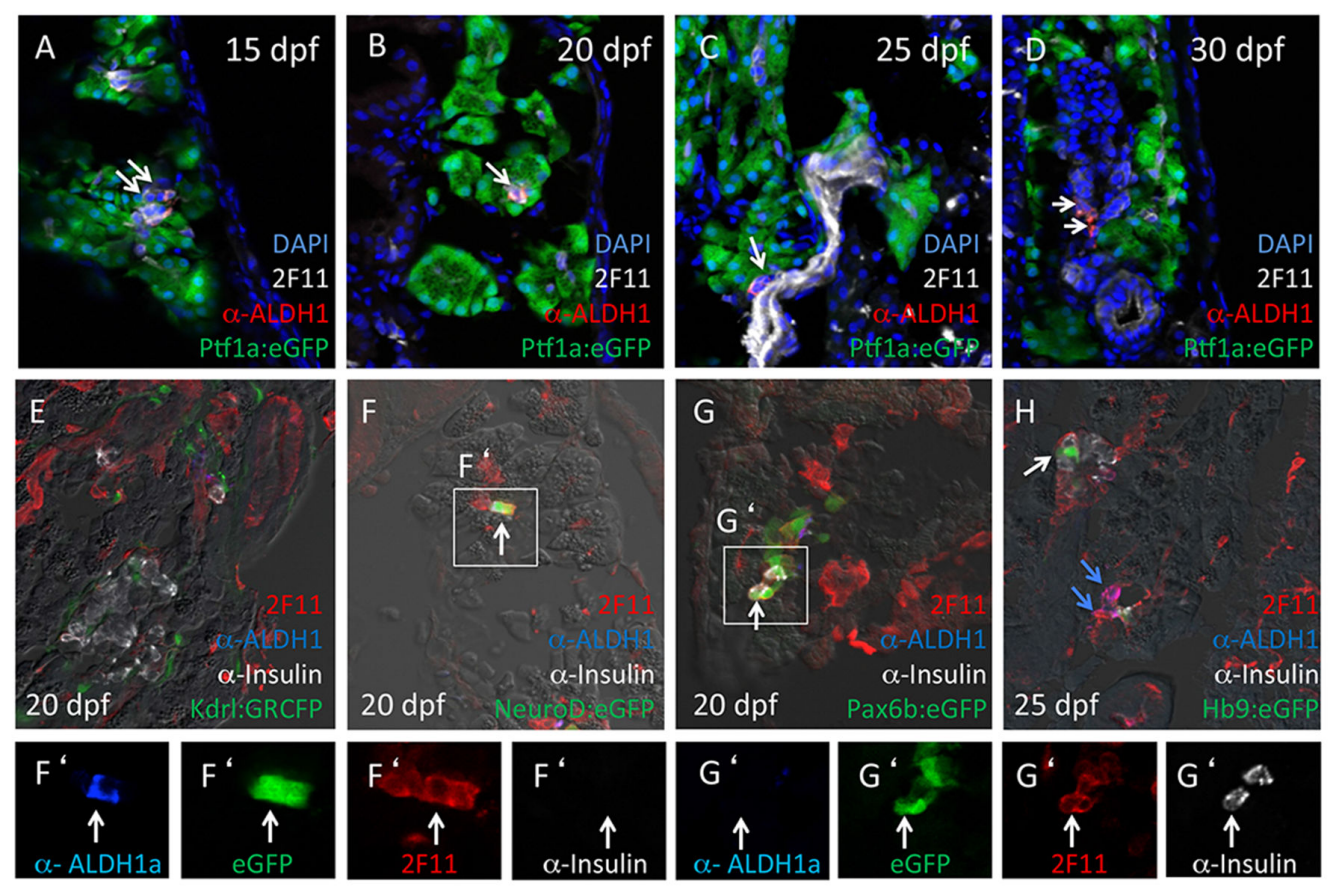
Aldh1-expressing cells express the pancreatic epithelial marker 2F11. (A-D) All Aldh1-expressing cells co-express the pancreatic epithelial marker 2F11 (white arrows). (E) 2F11 does not label GRCFP^pos^ vascular endothelial cells in Kdrl:GRCFP fish. (F-H) 2F11 labels both a subset of Insulin^pos^ cells (G and G’) and eGFP^pos^ cells in NeuroD:eGFP (F and F’), Pax6b:eGFP (G and G’) Hb9:eGFP (H) fish. White arrows in A-D indicate cells co-labeling for both 2F11 and Aldh1. White arrows in F and F’ indicate Insulin-negative cells co-expressing 2F11, Aldh1 and NeuroD:eGFP. White arrows in G and G’ indicate Aldh1-negative cell co-expressing Insulin, 2F11 and Pax6:eGFP. White arrow in H indicates Aldh1-negative cell expressing Insulin, 2F11 and Hb9:eGFP; blue arrows indicate Insulin-negative, Hb9:eGFP-negative cells expressing 2F11 and Aldh1.

**Figure 4:**
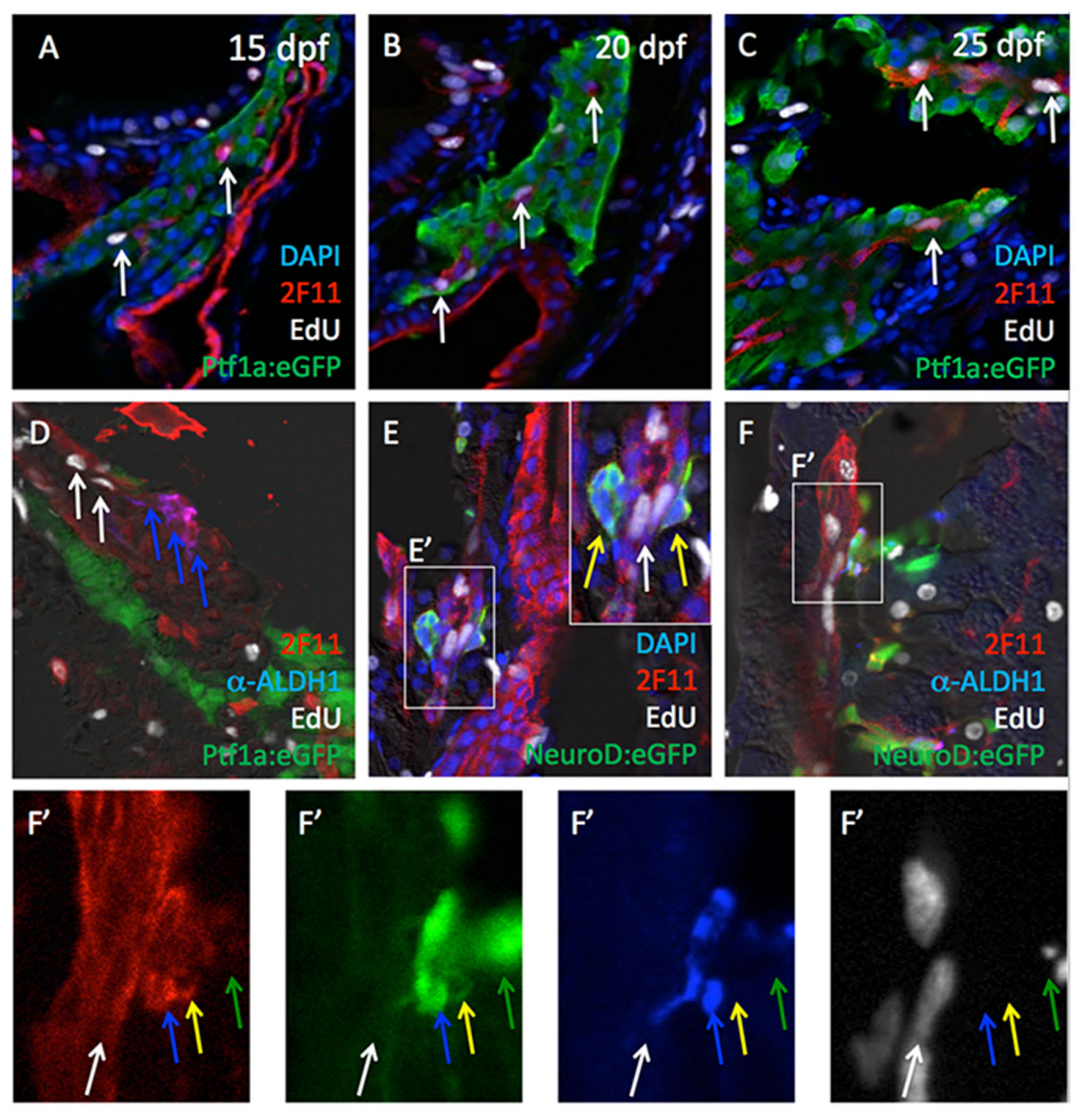
Cell proliferation among Aldh1- and 2F11-expressing cells. EdU signals (white arrows) were detected in subsets 2F11^pos^ cells at 15 (A), 20 (B) and 25 dpf (C). (D) Cells co-expressing Aldh1 and 2F11 (blue arrows) are not proliferative, but are localized in the adjacent to Aldh1-negative, EdU^pos^, 2F11^pos^ cells (white arrows). (E and E’) Cells co-expressing NeuroD and 2F11 (yellow arrows) are not proliferative but are localized adjacent to NeuroD:eGFP-negative, EdU^pos^, 2F11^pos^ cells (white arrows). (F and F’) Detailed geometry of 2F11, Aldh1, NeuroD:eGFP expression in association with EdU incorporation. White arrows indicate EdU ^pos^/2F11 ^pos^/Aldh1 ^neg^/NeuroD:eGFP^neg^ cells. Blue arrows indicate EdU^neg^ /2F11 ^pos^/Aldh1 ^pos^/NeuroD:eGFP^pos^ cells. Yellow arrows indicate EdU ^neg^/2F11 ^pos^/Aldh1 ^neg^/NeuroD:eGFP^pos^ cells. Green arrows indicate EdU ^neg^/2F11^neg^ /Aldh1 ^neg^/NeuroD:eGFP^pos^ cells.

Previous studies have suggested that, as in mammals, secondary islet formation in zebrafish originates from progenitor cells located within the forming ductal epithelium [10,20,21,34]. We therefore examined the relationship between Aldh1-positive, *neurod*-positive cells and labeling of 2F11, a marker of the forming pancreatic ductal epithelium [11,35,36]. These analyses revealed that all Aldh1-positive cells were also labeled with (Figure 3A-D). 2F11 also labeled Aldh1-negative, ptf1a:eGFP-negative tubular structures (Figure 3C), which were found to be distinct from vascular endothelium marked by kdrl:GRCFP (Figure 3E). In further evaluating the overlap between 2F11 labeling and the expression of Aldh1, neurod and insulin in larval zebrafish pancreas, we further observed that 2F11 also marked Aldh1-positive cells expressing *neurod* (Figure 3F and F’), as well as Aldh1-negative cells also expressing pax6b:GFP (Figure 3G and G’), insulin (Figure 3G and G’), and hb9 (Figure 3H). Using Edu incorporation to mark proliferating cells, we further identified a subset of 2F11 cells undergoing active proliferation (Figure 4A-F). However, EdU incorporation was limited to 2F11 cells not co-expressing Aldh1, *neurod* or *pax6b*, suggesting that commitment of 2F11 cells to the endocrine lineage is marked by relative or complete cell cycle exit. Further evaluation of the detailed spatial relationships between cells expressing the 2F11 epitope, Aldh1, *neurod* and *pax6b* suggested that proliferating 2F11 ^pos^/EdU ^pos^/Aldh1 ^neg^/neurod^neg^ cells were incorporated into tubular ductal epithelial structures, while non-proliferating 2F11 ^pos^/EdU ^neg^/Aldh1 ^pos^/neurod^pos^ endocrine progenitor cells appeared to delaminate directly from this epithelium (Figure 4F, F’), in a manner similar to that described in mammalian pancreas development.

### Aldh1-positive endocrine progenitor cells arise from Notch-responsive ductal progenitor cells

Within the emerging ductal epithelium, a subset of cells characterized by active Notch signaling are known to serve as multi-lineage pancreatic progenitor cells [10,21], referred to as pancreatic notch-responsive cells, or PNCs. We therefore sought to determine the relationship between PNCs, 2F11 labeling and Aldh1 expression in larval zebrafish pancreas. Using either Tg(*Tp1bglob:eGFP*)^um14^ *or* Tg(*Tp1bglob:mCherry*)^*jh11*^ to mark PNCs at 20 and 25dpf, we found that PNCs were 2F11-positive, and frequently incorporated EdU (Figure S1A-D). In contrast, we observed no overlap between Notch reporter expression and Aldh1 labeling (Figure S1E), suggesting that PNCs and Aldh1^pos^ cells represented distinct cell compartments. To further investigate potential overlap between Aldh1^pos^ cells and PNCs, we FACS sorted PNCs from Tg(*Tp1bglob:eGFP*)^*um14*^ adult zebrafish pancreas, and assessed Aldh1 expression following cytospin using immunofluorescent labeling. This revealed that Aldh1 expression was restricted to the eGFP^neg^, non-PNC population (Figure S2C, D). In contrast, Aldh1^pos^ cells sorted using the Aldefluor reagent [27] displayed strong immunoreactivity for Aldh1, further validating use of this Aldh1 antibody in zebrafish (Figure S2A, B). Additional analysis of gene expression using RT-PCR on FACS-sorted PNCs and FACS-sorted Aldh1^pos^ cells isolated from adult fish confirmed unique patterns of gene expression between these two subpopulations, with Aldh1^pos^ cells expressing high levels of *aldh1a2* and low levels of the general stem cell markers *cd133* and *sca1* (Figure S2 E), while PNCs expressed low levels of *aldh1a2*, high levels of *cd133* and moderate levels of *scal1* (Figure S2B). Together, these results suggest that the Aldh1^pos^ and PNC subpopulations represent distinct sets of endocrine progenitor cells.

**Figure 5:**
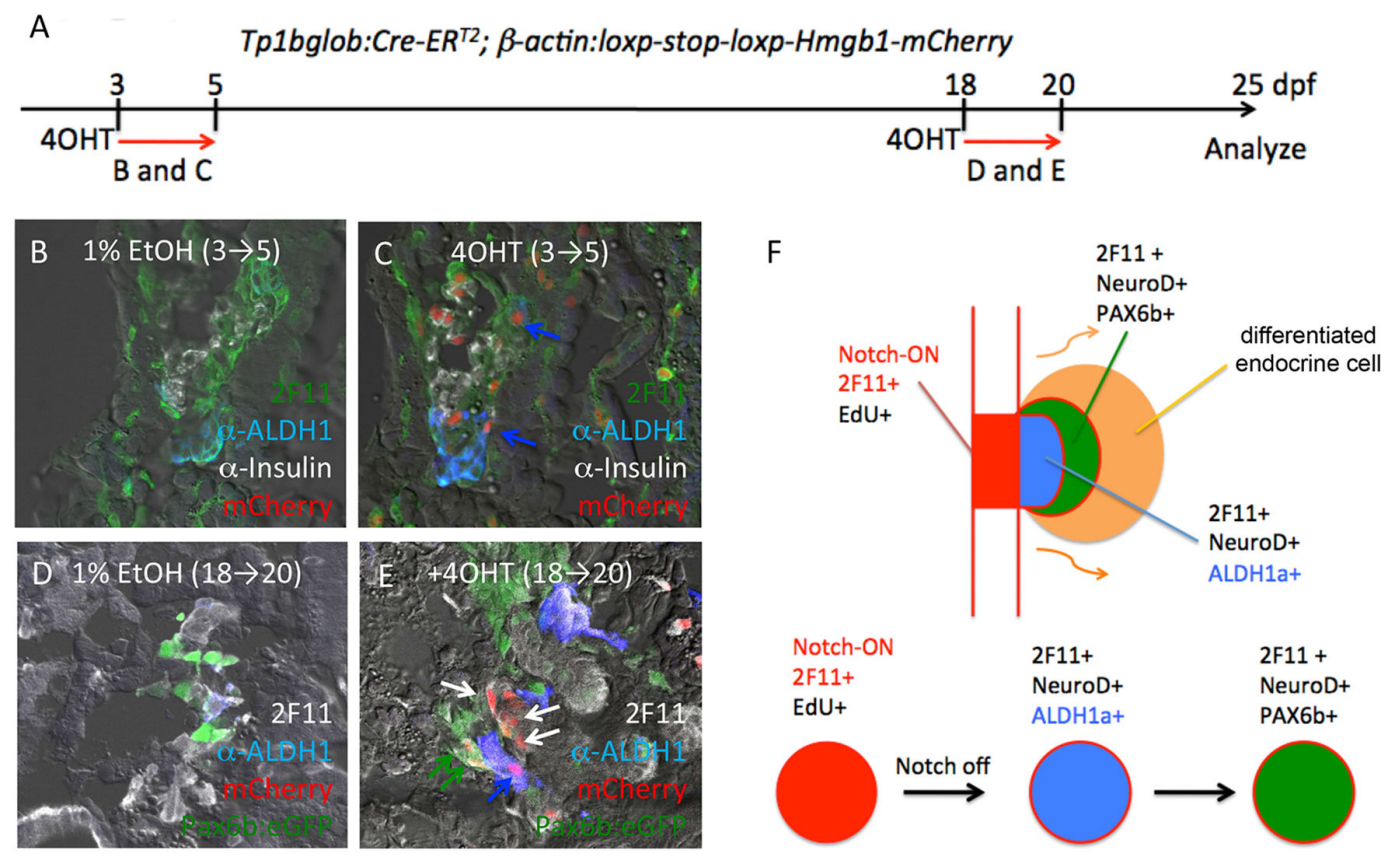
Aldh1-expressing cells are derived from Notch-responsive progenitor cells. (A) Schematic timeline for 4OHT-induced lineage labeling. Tp1bglob:CreER ^T2^/βactin:loxp-stop-loxp-hmgb-mCherry fish were treated with either 1% EtOH (B and D; negative controls) or with 4-hydroxy-tamoxifen (4OHT) between either 3-5 dpf (B and C) or 18-20 dpf (D and E), and then sacrificed for analysis at 25 dpf. Cells carrying the Tp1bglob:CreER^T2^ lineage label display red nuclei. (C) 2F11 ^pos^/Aldh1^pos^ cells arise from Notch-responsive cells labeled between 3-5 dpf (blue arrows). (E) Notch-responsive cells labeled between 18-20 dpf give rise to Aldh1^pos^ (blue arrows), Pax6:eGFP+ endocrine cells (green arrows) and ductal cells (white arrows). F, Schematic depiction of proposed lineage relationships between Notch-responsive progenitors (Notch-ON) and cells expressing 2F11, Aldh1, NeuroD and Pax6b during secondary islet formation. Notch-ON cells expressing 2F11 (red) give rise to Notch-OFF, 2F11^pos^, NeuroD^pos^, Aldh1^pos^ cells (blue), which then inactivate Aldh1 and activate Pax6 expression (green) prior to undergoing terminal endocrine differentiation (orange).

In order to determine whether Notch activation and Aldh1 expression might represent sequential steps in pancreatic progenitor cell differentiation, we next performed Cre/lox based lineage tracing of the PNC lineage, as previously described [21]. For these studies, we utilized the *Tg*(*Tp1glob:creERT2*)^*jh12*^ and *Tg*(βactin*:loxP-stop-loxP-hmgb1-mCherry*)^*jh15*^ alleles, and transiently activated Cre activity using 4-hydroxy-tamoxifen (4OHT) at either 3-5 dpf or 18-20 dpf, followed by harvest at 25dpf (Figure 5A). These studies confirmed that PNCs labeled at either 3-5 dpf or 18-20 dpf gave rise not only to later appearing endocrine cells and ductal cells as previously reported, but also gave rise to Aldh1-positive cells (Figure 5B-E), allowing us to order PNCs and Aldh1-positive, *neurod*-positive endocrine progenitor cells as sequential populations arising during endocrine differentiation. Together with the spatial labeling patterns observed for Aldh1, *neurod* and *pax6b* expression as described above, these data suggest a step-wise model of endocrine differentiation in the formation of zebrafish secondary islet formation, as depicted schematically in Figure 5F.

### Inhibition of Aldh1 enzymatic activity increases the number of Aldh1^pos^ endocrine progenitor cells and induces their premature differentiation

**Figure 6:**
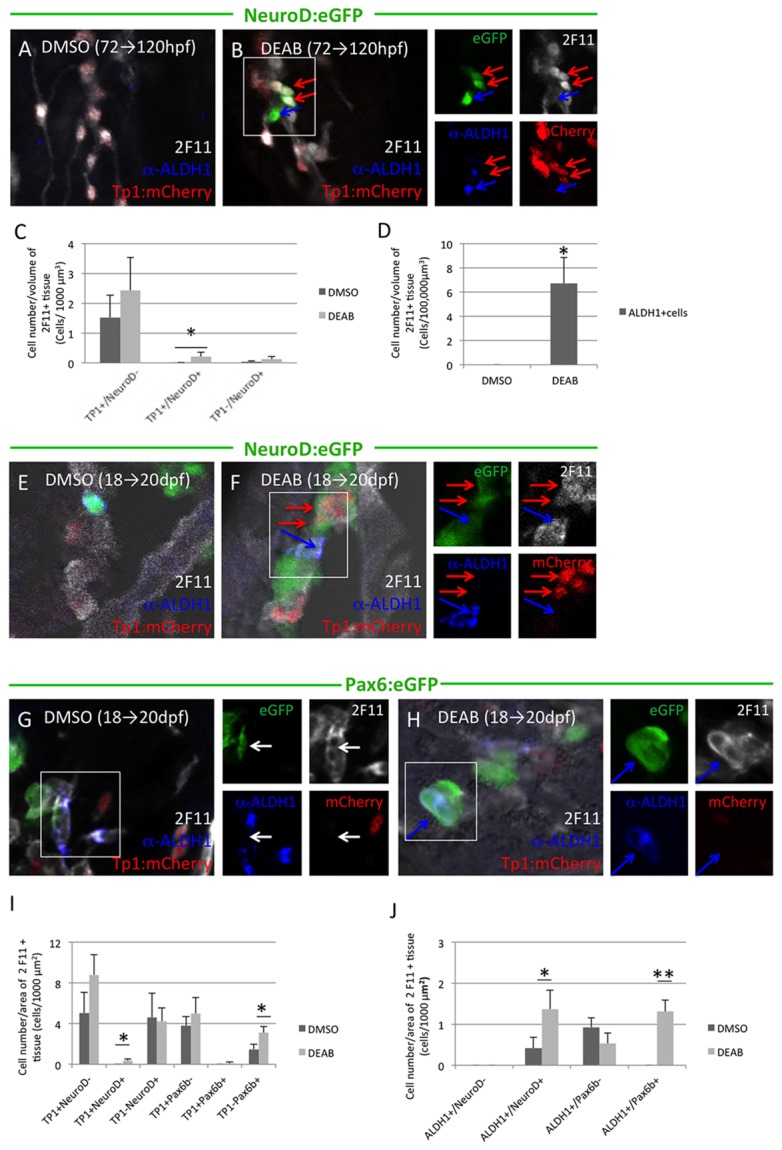
Inhibition of Aldh1 enzymatic activity increases the number of Aldh1- and NeuroD-expressing endocrine progenitor cells and induces their premature differentiation. Panels to the right of merged images represent corresponding single channel images. (A and B) NeuroD:eGFP/Tp1:hmgb-mCherry fish were treated with DMSO (A) or DEAB (B) at 72-120 hpf. DEAB treatment increased the number of Notch-responsive cells also expressing NeuroD:eGFP (red arrows). (C) Quantification of the number of eGFP^pos^ and/or mCherry^pos^ cells in 1000 µm^3^ of 2F11^pos^ tissue in NeuroD:eGFP/Tp1:hmgb-mCherry fish treated with DMSO or DEAB between 72-120 hpf. Inhibition of Aldh1a induced NeuroD:eGFP expression in Notch-responsive cells. Values indicate mean + SEM, with minimum of 20 larvae analyzed for each condition. (*) indicates p<0.05. (D) Quantification of the number of Aldh1-expresing cells in 100,000 µm^3^ of 2F11^pos^ tissue in NeuroD:eGFP/Tp1:hmgb-mCherry fish treated with DMSO or DEAB treatment between 72-120 hpf. Values indicate mean + SEM, with minimum of 20 larvae analyzed for each condition. (*) indicates p<0.05. (E and F) NeuroD:eGFP/Tp1:hmgb-mCherry fish were treated with DMSO (E) or DEAB (F) at 18-20 dpf. DEAB treatment increased the number of Notch-responsive cells also expressing NeuroD:eGFP (red arrows), and these cells were frequently adjacent to Aldh1-expressing cells (blue arrows). (G and H) Pax6b:eGFP/Tp1:hmgb-mCherry fish were treated with DMSO (G) or DEAB (H) between 18-20 dpf. DEAB treatment activated Pax6:eGFP expression in Aldh1-expressing cells. (I) Quantification of number of eGFP^pos^ and/or mCherry^pos^ cells in 1000 µm^2^ of 2F11^pos^ tissue in NeuroD:eGFP/Tp1:hmgb-mCherry or Pax6b:eGFP/Tp1:hmgb-mCherry fish treated with DMSO or DEAB between 18-20 dpf. NeuroD:eGFP, but not Pax6:eGFP expression is activated within Notch responsive cells following DEAB treatment. In contrast, Pax6:eGFP but not NeuroD:eGFP expression is induced in cells not expressing Tp1:hmgb1-mCherry. (*) indicates p<0.05, with n=8 fish per condition and a minimum of 3 sections analyzed per fish. (J) Quantification of Aldh1-expressing cells also expressing either NeuroD:eGFP or Pax6:eGFP following treatment with either DMSO or DEAB between 18-20 dpf. DAEB treatment increases the number of cells Aldh1-expressing cells also expressing either NeuroD:eGFP or Pax6:eGFP. (*) and (**) indicates p<0.05 and p<0.01, respectively. n=10 fish per condition and a minimum of three sections analyzed per fish.

Prior studies have demonstrated that, in addition to exerting a positive influence on the early pancreatic progenitor field [22-24], Aldh1 enzymatic activity and associated retinoic acid production exert a negative influence on β-cell differentiation during secondary islet formation [25]. We therefore examined how inhibition of Aldh1 enzymatic activity influenced Aldh1 cell behavior in conjunction with Notch signaling and neurod expression in larval *Tg*(*Tp1bglob:hmgb1-mCherry*)^*jh11*^ *; Tg*(*neurod:eGFP*) zebrafish pancreas. Using the Aldh1 inhibitor DEAB, we observed that inhibition of Aldh1 enzymatic activity between 72 hpf and 120 hpf was associated not only with premature activation of *neurod* expression in PNCs, but also with a marked increase in the number of Aldh1-positive cells (Figure 6A-D). When Aldh1 enzymatic activity was inhibited between 18 and 20 dpf, a similar increase in *neurod* expression was observed in PNCs (Figure 6E, F and I). Pax6b-expressing cells also increased in number, although pax6b expression was confined to cells not expressing Tp1:mCherry (Figure 6I). Activation of not only neurod, but also pax6b expression was observed in Aldh1-positive cells (Figure 6E, F and J), suggesting an additional negative influence of Aldh1 enzymatic activity on the further differentiation of Aldh1-positive, *neurod*-positive endocrine progenitor cells.

## Discussion

In this study, we have identified a low-abundance population of Aldh1-expressing cells in larval zebrafish pancreas. These cells increase in abundance in association with secondary islet formation and are frequently located within the pancreatic ductal epithelial tree adjacent to insulin expressing cells, consistent with possible endocrine progenitor function. This conclusion is further supported by the fact that Aldh1-expressing cells co-express the endocrine progenitor marker *neurod*, but do not normally co-express either *insulin, pax6* or other genes typically expressed in more mature endocrine cells. In addition, their abundance is significantly increased by inhibition of Aldh1 enzymatic activity, a manipulation known to promote the precocious differentiation of pancreatic progenitor cells and the acceleration of secondary islet formation [25]. Using formal Cre/lox lineage tracing, we also show that, like differentiated endocrine cells [21], Aldh1-expressing cells are the progeny of pancreatic notch-responsive progenitor cells (PNCs) located within the forming ductal system of the larval zebrafish pancreas. In addition to accelerating the formation of Aldh1-expressing cells from PNCs, inhibition of Aldh1 enzymatic activity using DEAB results in the premature activation of pax6 expression among cells expressing Aldh1.

These observations suggest that the increase in secondary islet formation observed following inhibition of Aldh1 enzymatic activity is associated with the premature activation of *neurod* and Aldh1 expression among PNCs, as well as the accelerated maturation of Aldh1-positive, *neurod*-positive cells, reflected by activation of *pax6b* expression. These findings place Aldh1-positive, *neurod*-positive cells as a critical intermediate between PNCs and more differentiated endocrine cells, and further suggest that Aldh1 enzymatic activity may play an important functional role in regulating both entry to and exit from this intermediate progenitor compartment.

**Figure 7:**
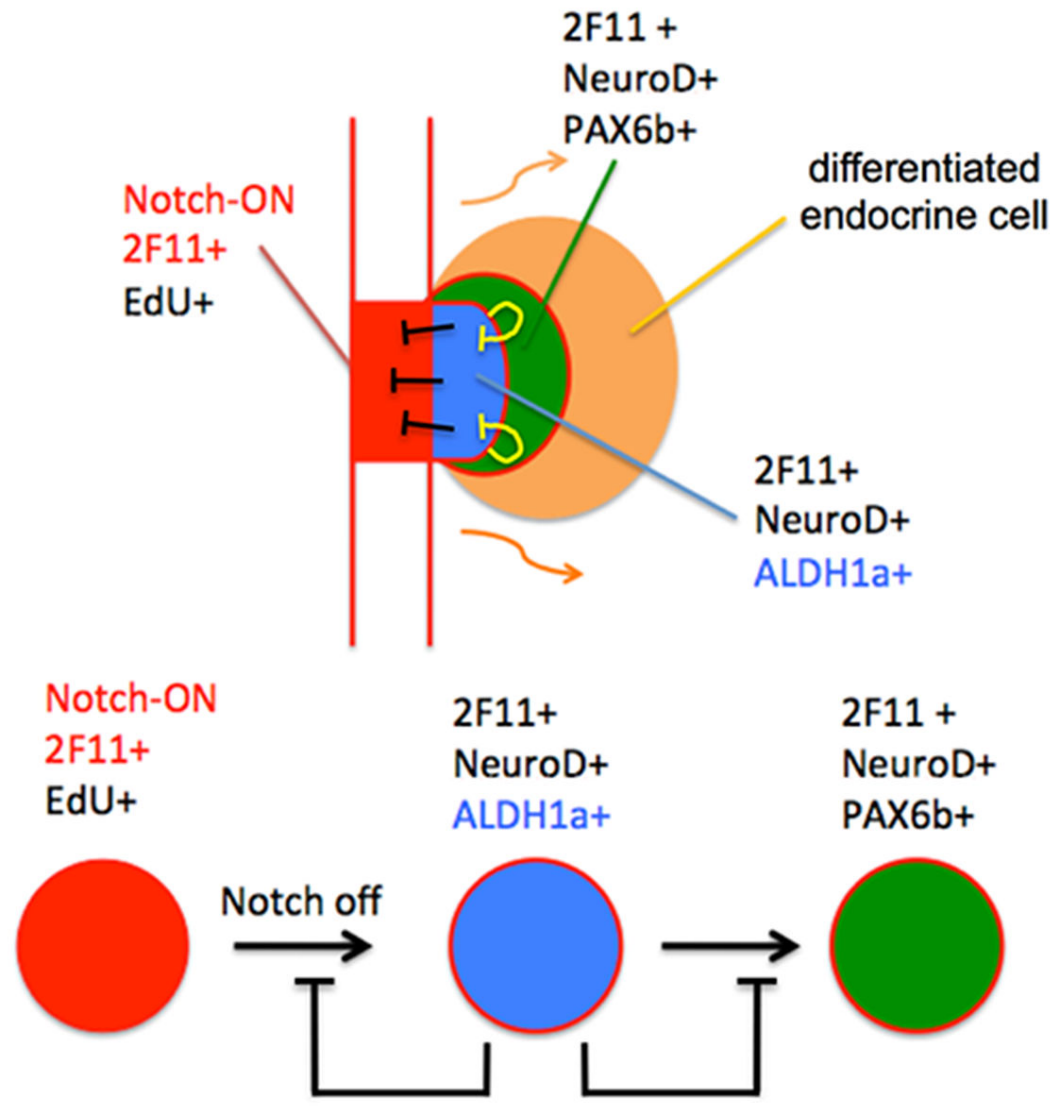
Proposed model for the role of Aldh1-expressing cells in zebrafish secondary islet formation. Notch-ON cells expressing 2F11 (red) give rise to Notch-OFF, 2F11^pos^, NeuroD^pos^ Aldh1^pos^ cells (blue), which then inactivate Aldh1 and activate Pax6 expression (green) prior to undergoing terminal endocrine differentiation (orange). Aldh1 enzymatic activity and associated retinoic acid production inhibit the progression of Notch-ON cells into the NeuroD^pos^, Aldh1^pos^ endocrine progenitor pool, as well as the progressive differentiation of NeuroD^pos^, Aldh1^pos^ cells.

Based on these observations, we now propose a model of zebrafish secondary islet formation, in which retinoic acid plays an integral role in controlling the progression of pancreatic progenitor cells to a differentiated endocrine phenotype (Figure 7). During secondary islet formation, pancreatic epithelial expression of Aldh1 is initiated by former PNCs as they inactivate notch signaling, delaminate and start to express early markers of the endocrine lineage. As these cells continue to differentiate, they switch off Aldh1 expression and turn on markers of more mature endocrine cells, including *pax6b* and *insulin*. Blocking production of retinoic acid accelerates this process. We hypothesize that retinoic acid is produced following the activation of Aldh1 expression immediately following delamination from ductal epithelium and commitment to the endocrine lineage. Retinoic acid produced by these cells then acts to prevent additional cells from delaminating, and also impedes the further maturation of cells that have already entered the endocrine progenitor pool. This creates an effective rheostat governing the ultimate number of endocrine cells.

These findings contribute to recent heightened awareness of Aldh1 expression and enzymatic activity as markers and possible regulators of pancreatic progenitor cells in adult and embryonic pancreas. In embryonic mouse pancreas, Aldh1 is expressed in “tip” progenitor cells, a zone known to harbor multi-lineage endocrine and exocrine progenitor cells [26,27]. In murine ES cell-derived pancreatic progenitor cells, activation of an inducible Ngn3 transgene upregulated expression of both *NeuroD* and the Aldh1 isoform *Aldh1b1* [37], potentially echoing our findings regarding *neurod* and Aldh1 coexpression in zebrafish pancreatic progenitor cells. As in zebrafish [25], inhibition of Aldh1 enzymatic activity in developing mouse pancreas is associated with precocious endocrine differentiation [26]. In adult mouse pancreas, epithelial expression of Aldh1 is largely confined to centroacinar and terminal duct cells [27]. When isolated based upon high-level Aldh1 enzymatic activity, these adult cells display both endocrine and exocrine progenitor capacities *in vitro*, and are also uniquely able to contribute to the endocrine and exocrine lineages of the embryonic pancreas [27]. Thus the expression of Aldh1 isoforms appears to be a common feature of pancreatic progenitor cells in both mouse and zebrafish.

It is important to acknowledge that many aspects of our model remain speculative. With respect to our hypothesis that zebrafish Aldh1-expressing cells serve as direct precursors to the *pax6* lineage, it has proven difficult to detect significant numbers of Aldh1-expressing cells prior to the onset of pax6:eGFP expression. This may reflect the fact that, under normal conditions, there is a very narrow temporal window of Aldh1 expression during endocrine differentiation; alternatively, it may reflect differential sensitivities in the assessment of gene and protein expression using antibodies vs. transgenic reporters. It also remains unclear whether all endocrine cell types emanate from progenitor cells expressing both Aldh1 and *neuroD*. This point is especially relevant given our finding that many *neuroD*-positive cells in larval zebrafish pancreas do not co-express Aldh1, as well as recent observations in the mouse suggesting that early pancreatic progenitor cells may already be compartmentalized into unipotent subpopulations [38]. In the mouse, adult pancreatic Aldh1-expressing cells are capable of generating both insulin-expressing cells and glucagon-expressing cells [27]; whether Aldh1-expressing cells in larval zebrafish pancreas can similarly serve as precursors for multiple endocrine cell types remains unknown.

In addition, while inhibition of Aldh1 enzymatic activity is associated with precocious endocrine differentiation in both mouse and zebrafish, the specific products of Aldh1 enzymatic activity responsible for mediating this effect remain unknown. While Aldh1 enzymatic activity may be linked to retinoic acid synthesis, the reagents currently utilized to isolate cells based on Aldh1 expression do not specifically test for retinal dehydrogenase activity. In addition, embryonic and adult mouse pancreatic cells marked by Aldh1 immunoreactivity have been shown to express some Aldh1 isoforms that carry retinal dehydrogenase activity (e.g. *Aldh1a1*), and some isoforms (e.g. *Aldh1a7, Aldh1B1*) lacking this activity [26,27]. While we have shown that the zebrafish Aldh1 epitopes recognized by Aldh1 immunostaining are indeed associated with Aldh1 enzymatic activity (Figure S2), the precise zebrafish Aldh1 isoforms recognized by currently available Aldh1 antibodies are unknown, further underscoring the difficulties in determining the relevance of retinoic acid production by these cells.

Together with prior reports [25], our findings suggest that Aldh1 enzymatic activity may serve as a critical gatekeeper of endocrine lineage commitment and maturation, and that pharmacologic inhibition of this activity may play a role in facilitating β-cell regeneration.

## Supporting Information

Figure S1 **Aldh1-expressing cells are distinct from Notch-responsive cells.** (A, A’, B and B’) Notch responsive cells (Tp1:mCherry; white arrows) also express 2F11 at 20 (A and A’) and 25 dpf (B and B’). (C, C’, D and D’) Cells co-expressing Tp1:eGFP and 2F11^pos^ are proliferative (white arrows). (E) At 20 dpf, Aldh1^pos^ cells (blue arrows) are a distinct from Notch-responsive cells (red arrows), but are sometimes localized adjacent to Notch-responsive cells in the ductal epithelium. All images acquired from fish at 20-25dpf.
(TIF)

Figure S2 **Aldh1-expressing cells and Notch-responsive cells represent distinct cell types in adult zebrafish pancreas.** (A-D) FACS sorting was performed on single cells isolated from adult pancreas of wild type (A and B) or Tp1:eGFP (C and D) fish. Wild-type cells were labeled with Aldefluor and then FACS sorted, while cells from Tp1:eGFP fish were sorted for eGFP. Sorted populations were then subjected to cytospin and immunofluorescent labeling for Aldh1. In the case of cells from Tp1:eGFP fish, labeling was also performed for eGFP. Note that Aldh1 protein is detected in Aldefluor^pos^ but not Tp1:eGFP^pos^ cells, while eGFP is detected in Tp1:eGFP^pos^ but not Aldefluor^pos^ cells. Arrows in (C) indicate low-abundance Aldh1-sorted cells present in Tp1:eGFP^neg^ but not Tp1:eGFP^pos^ cell fraction. E, RT-PCR analysis of gene expression in FACS sorting populations. Positive and negative populations of cells sorted for either Aldh1 activity or Tp1:eGFP expression displayed differential expression of *aldh1a2*, *prom1*, and *sca1*, with contrasting patterns of enrichment in Aldefluor^pos^ vs. Aldefluor^neg^ and Tp1:eGFP^pos^ vs. Tp1:eGFP^neg^ cell fractions, further documenting the non-overlapping nature of Aldh1-expressing and Notch-responsive progenitor cells. Note that both populations are depleted of transcripts encoding differentiated endocrine (insulin), acinar (elastase1) and ductal (krt18) markers.
(TIF)
