# Supplementary figures and images for: Aldh1-Expressing Endocrine Progenitor Cells Regulate Secondary Islet Formation in Larval Zebrafish Pancreas
Source: PLoS One. 2013 Sep 17;8(9):e74350. doi: 10.1371/journal.pone.0074350 (PMC3798260; doi:10.1371/journal.pone.0074350)

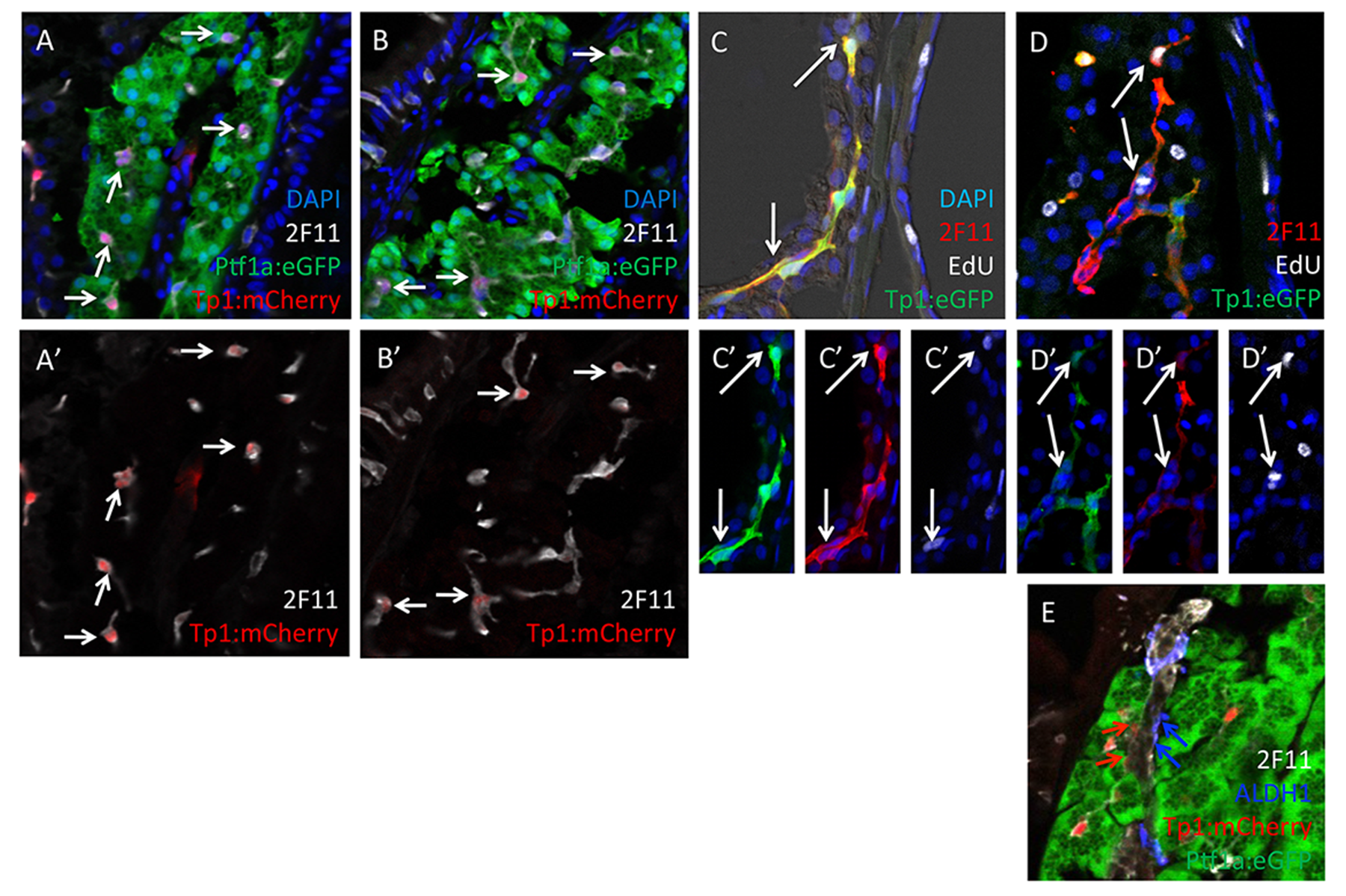

Supplement: Figure S1 — Aldh1-expressing cells are distinct from Notch-responsive cells. (A, A’, B and B’) Notch responsive cells (Tp1:mCherry; white arrows) also express 2F11 at 20 (A and A’) and 25 dpf (B and B’). (C, C’, D and D’) Cells co-expressing Tp1:eGFP and 2F11pos are proliferative (white arrows). (E) At 20 dpf, Aldh1pos cells (blue arrows) are a distinct from Notch-responsive cells (red arrows), but are sometimes localized adjacent to Notch-responsive cells in the ductal epithelium. All images acquired from fish at 20-25dpf. (TIF) [file pone.0074350.s001.tif]

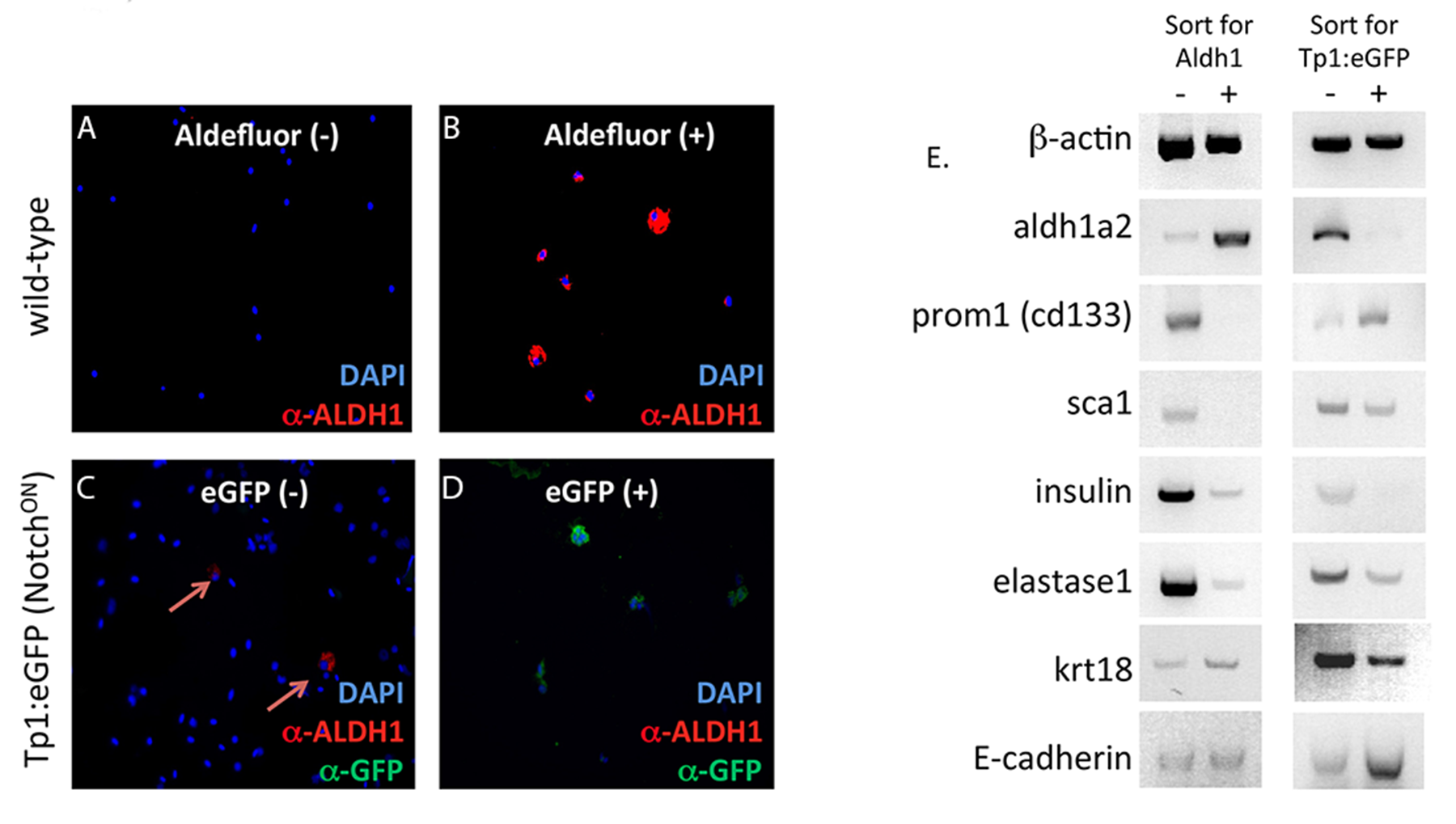

Supplement: Figure S2 — Aldh1-expressing cells and Notch-responsive cells represent distinct cell types in adult zebrafish pancreas. (A-D) FACS sorting was performed on single cells isolated from adult pancreas of wild type (A and B) or Tp1:eGFP (C and D) fish. Wild-type cells were labeled with Aldefluor and then FACS sorted, while cells from Tp1:eGFP fish were sorted for eGFP. Sorted populations were then subjected to cytospin and immunofluorescent labeling for Aldh1. In the case of cells from Tp1:eGFP fish, labeling was also performed for eGFP. Note that Aldh1 protein is detected in Aldefluorpos but not Tp1:eGFPpos cells, while eGFP is detected in Tp1:eGFPpos but not Aldefluorpos cells. Arrows in (C) indicate low-abundance Aldh1-sorted cells present in Tp1:eGFPneg but not Tp1:eGFPpos cell fraction. E, RT-PCR analysis of gene expression in FACS sorting populations. Positive and negative populations of cells sorted for either Aldh1 activity or Tp1:eGFP expression displayed differential expression of aldh1a2, prom1, and sca1, with contrasting patterns of enrichment in Aldefluorpos vs. Aldefluorneg and Tp1:eGFPpos vs. Tp1:eGFPneg cell fractions, further documenting the non-overlapping nature of Aldh1-expressing and Notch-responsive progenitor cells. Note that both populations are depleted of transcripts encoding differentiated endocrine (insulin), acinar (elastase1) and ductal (krt18) markers. (TIF) [file pone.0074350.s002.tif]
